# Construction of rGO/WO_3_/SiNWs Ternary Composite Structure and Its Low-Temperature NO_2_ Gas-Sensing Performance

**DOI:** 10.3390/mi17101170

**Published:** 2026-10-08

**Authors:** Fengyun Sun, Encheng Zhang, Zhihai Wang

**Affiliations:** 1 College of Mechanical and Electrical, China University of Petroleum-Beijing at Karamay, Karamay 834000, China; 2 Heilongjiang Provincial Key Laboratory of Micro-nano Sensitive Devices and Systems, Heilongjiang University, Harbin 150080, China; encheng.zhang@zymec.cn; 3 College of Petroleum Engineering, China University of Petroleum-Beijing at Karamay, Karamay 834000, China

**Keywords:** rGO, WO_3_, SiNWs, heterojunction, NO_2_ gas sensing

## Abstract

To address the issues of low sensitivity and high operating temperature in silicon nanowire (SiNWs)-based NO_2_ sensors, this study constructs a ternary rGO/WO_3_/SiNWs heterostructure by spin-coating reduced graphene oxide/tungsten oxide (rGO/WO_3_) composites onto SiNWs surfaces. Systematic characterizations (XRD, XPS, SEM, TEM) confirm the formation of intimate multiple p–n heterojunctions among WO_3_ nanorods, rGO sheets, and the SiNWs scaffold. Gas-sensing tests reveal that the rGO doping level critically modulates the sensor performance: the 0.4 wt% rGO-modified sample exhibits a sensitivity of 48.3 toward 1 ppm NO_2_ at 127 °C with response/recovery times of 16.4/244.8 s, while the 2 wt% rGO-modified sample lowers the optimal operating temperature to 101 °C and shortens the response/recovery times significantly to 4/184.8 s. Mechanistic analysis attributes the enhanced performance to the synergistic modulation of interfacial depletion layers and carrier transport via multiple heterojunctions among WO_3_, rGO, and SiNWs, in which the rGO conductive network accelerates charge transfer. This work provides an experimental basis for heterojunction engineering of high-performance, low-power NO_2_ sensors.

## 1. Introduction

Silicon nanowires (SiNWs) combine a high surface-to-volume ratio, efficient carrier transport, and compatibility with CMOS and VLSI technologies, making them attractive for gas sensing [1,2,3]. Nonetheless, bare SiNWs still exhibit limited sensitivity and selectivity toward oxidizing gases such as NO_2_, and thus typically require surface functionalization to improve their sensing performance [4,5,6].

Among various metal oxide functional materials such as SnO_2_ [7], ZnO [8,9,10,11], and WO_3_, WO_3_ stands out as a typical n-type transition metal oxide and exhibits excellent specific recognition capability toward NO_2_ [12,13,14,15,16]. Our previous work demonstrated that the WO_3_/SiNWs composite structure could reduce the optimal operating temperature to 127 °C [17]; nevertheless, its relatively high intrinsic resistance and still-elevated operating temperature limit its further application in low-power scenarios. Reduced graphene oxide (rGO), featuring outstanding electrical conductivity and a two-dimensional lamellar structure, can serve as an efficient electron transfer channel and active material support [18,19,20,21]. When WO_3_ is combined with rGO, a p–n heterojunction is established at the interface, which modulates band bending and the interfacial depletion layer width, thereby significantly affecting carrier separation and transport processes [22,23,24,25].

Although the WO_3_/SiNWs binary system has shown promising potential, a systematic experimental investigation into how rGO incorporation quantitatively affects the interface barrier height, depletion layer width, and the overall gas-sensing kinetics is still lacking. In this context, we further integrated the rGO/WO_3_ composite with SiNWs to construct a ternary rGO/WO_3_/SiNWs hybrid system, aiming to achieve significantly enhanced NO_2_ sensing performance through synergistic effects among the components, including heterojunction barrier modulation, increased active adsorption sites, and optimized charge transfer pathways.

In this work, ternary rGO/WO_3_/SiNWs composites with different rGO doping levels were prepared. Their phase composition, morphology, and surface chemical states were characterized, and the effect of rGO content on the NO_2_ sensing temperature, sensitivity, response/recovery kinetics, and detection limit was examined. The origin of the enhanced gas-sensing performance in this ternary system is discussed in terms of band theory, aiming to provide guidance for the rational design of high-performance NO_2_ sensors operating at low temperatures (101–127 °C).

## 2. Materials and Methods

### 2.1. Fabrication of rGO/WO_3_/SiNWs Composite Gas Sensors

Rectangular strips (25 mm × 5 mm) were prepared by dicing single-side-polished p-type Si(100) wafers (resistivity: 0.001–0.005 Ω·cm; diameter: 100 ± 0.2 mm; thickness: 525 ± 25 μm). To remove dust, organic contaminants, and the native oxide layer, the as-cut strips were thoroughly cleaned prior to etching.

**Figure 1:**
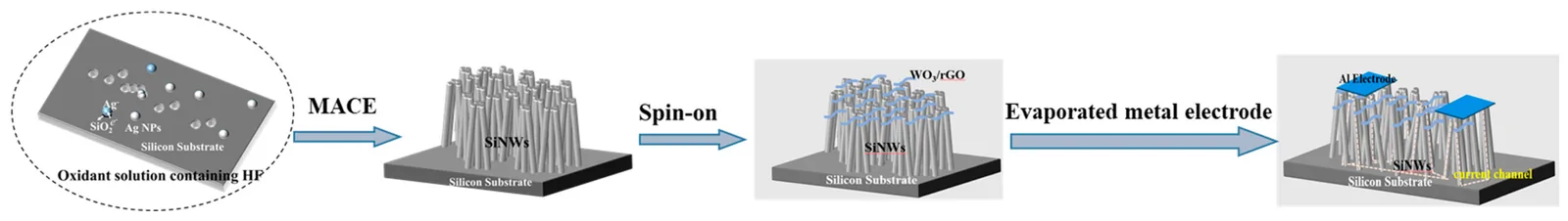
Schematic of the rGO/WO_3_/SiNWs composite gas sensor fabrication.

The fabrication procedure of the rGO/WO_3_/SiNWs composite gas sensors is depicted in Figure 1. SiNWs were prepared by two-step metal-assisted chemical etching (MACE). Cleaned Si(100) substrates were immersed in HF (40 wt%, 10 mL) and deionized water (10 mL) for 60 s, rinsed, and then soaked in a deposition solution of 4.4 M HF and 0.01 M AgNO_3_ for 60 s with the polished side facing up. After rinsing, the substrates were etched in 4.4 M HF and 0.8 M H_2_O_2_ for 1 h, immersed in 68% HNO_3_ for 60 s to remove residual Ag, rinsed, and dried.

WO_3_ nanorods were synthesized hydrothermally. Na_2_WO_4_·2H_2_O (0.008 mol) was dissolved in deionized water (50 mL) under stirring at 300 rpm, followed by addition of K_2_SO_4_ at a molar ratio of 200% relative to Na_2_WO_4_·2H_2_O. The pH was adjusted to 1.5 with 6 M HCl under stirring at 800 rpm for 15 min. The mixture was transferred to a Teflon-lined autoclave and heated at 180 °C for 12 h. The precipitate was collected by centrifugation, washed three times with deionized water and ethanol, dried at 80 °C, ground, and calcined at 500 °C for 2 h (5 °C/min).

For the composite, a 2.5 mg/mL GO dispersion was prepared by dispersing 0.01 g of GO in 4 mL of deionized water under ultrasonication for 1 h. Three portions of 0.19 g WO_3_ were each added to 15 mL of methanol and ultrasonicated for 30 min. GO dispersion was then added dropwise to achieve initial GO/WO_3_ mass ratios of 0.2, 0.4, and 2 wt%. These values refer to the initial mass ratios of GO to WO_3_ in the precursor suspension, not to the measured rGO content in the deposited films. After further ultrasonication for 30 min, the supernatant was collected by centrifugation (3000 rpm, 10 min) and spin-coated onto SiNWs at 1500 r/min for five cycles (30 s per cycle, 20 μL per cycle). The samples were labeled S-1, S-2, and S-3, dried at 60 °C for 1 h, and annealed at 300 °C for 2 h in N_2_ to reduce GO to rGO.

Finally, two square Al electrodes (4 mm × 2 mm, 12 mm apart, ~2 μm thick) were deposited by vacuum evaporation, thereby completing the rGO/WO_3_/SiNWs composite structure.

### 2.2. Materials Characterization

The crystal structure, morphology, and surface chemical states of the as-prepared materials were characterized by XRD (D8 Advance, Bruker, Karlsruhe, Germany, Cu Kα), SEM (S-3400, Hitachi, Tokyo, Japan), TEM (Talos F200X G2, Thermo Fisher Scientific, Waltham, MA, USA), and XPS (Escalab 250Xi, Thermo Fisher Scientific, Al Kα), respectively.

The NO_2_-sensing characteristics of the rGO/WO_3_/SiNWs composite were investigated using a gas-sensing setup, which is reported elsewhere [11]. The gas-sensing setup comprised a multimeter (DM3058E, Rigol, Suzhou, China), a measurement circuit, and a host computer. A 20 L sealed chamber was held at 36 RH%, with the gas sensor temperature controlled by an HP-E1010 heating stage. The resistance variation with time was measured by the DM3058E multimeter. NO_2_ was injected into the chamber by syringe (static gas distribution), and the chamber was opened for air recovery once the sensor resistance stabilized. The response is defined as *R*_a_/*R*_g_, where *R*_a_ and *R*_g_ are the baseline resistance in air and the steady-state resistance in NO_2_, respectively. Response time was defined as the time to reach 90% of the steady-state resistance upon exposure to the NO_2_ target gas, and recovery time as the time to return to 90% of the steady-state value in air after NO_2_ gas removal.

## 3. Results

### 3.1. Structural and Morphological Characteristics of the rGO/WO_3_/SiNWs Composite

**Figure 2:**
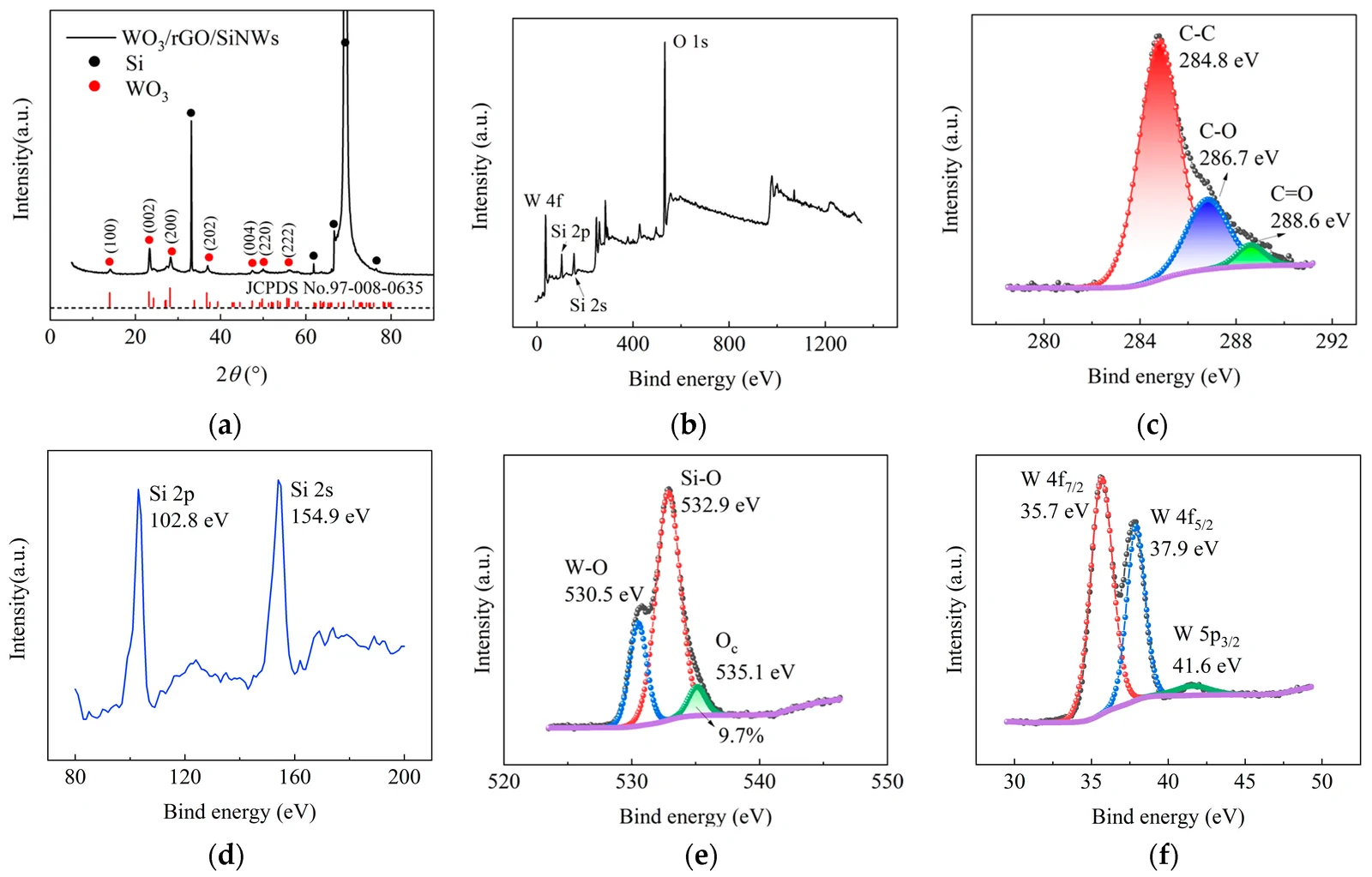
XRD patterns and XPS spectra of the rGO/WO_3_/SiNWs composite. (**a**) XRD pattern; (**b**) XPS survey spectrum; high-resolution XPS spectra of (**c**) C 1s, (**d**) Si 2p and Si 2s, (**e**) O 1s, and (**f**) W 4f.

The crystal structure and surface chemical states of the rGO/WO_3_/SiNWs composite were examined by XRD and XPS (Figure 2). In the XRD pattern (Figure 2a), characteristic diffraction peaks of Si are observed at 2θ values of 33.1°, 61.9°, 66.7°, 69.3°, and 76.5°. The dominant peak near 69.1° originates from the Si(400) diffraction of the single-crystalline Si(100) substrate, and its high intensity confirms that the substrate is highly oriented. The additional peaks at 33.1°, 61.9°, 66.7°, and 76.5° are attributed to lattice distortion, defects, or residual stress introduced into the Si crystal during the metal-assisted chemical etching (MACE) process for SiNWs formation. These structural imperfections can activate diffraction from other crystallographic planes that are normally absent or very weak in pristine single-crystalline Si.

The additional peaks located at 14.1°, 23.3°, 28.3°, 37°, 47.5°, 49.9°, and 56.1° can be indexed to the (100), (002), (200), (202), (004), (220), and (222) planes of hexagonal WO_3_ (JCPDS 97-008-0635), respectively. The relatively weak intensity of the WO_3_ diffraction peaks compared to those of Si is likely attributable to the low WO_3_ content or the thin film thickness. No diffraction peaks corresponding to rGO are detected, which may be due to the close attachment of rGO to the (102) plane of WO_3_ and its relatively low content.

The XPS survey spectrum (Figure 2b) reveals only Si, C, W, and O in the sample. The C 1s spectrum (Figure 2c) is fitted with three peaks at 284.8, 286.7, and 288.6 eV, corresponding to C–C, C–O [20], and C=O [21], respectively. Si 2p and Si 2s signals appear at 102.8 and 154.9 eV (Figure 2d). The O 1s spectrum (Figure 2e) shows three components at 530.5, 532.9, and 535.1 eV, ascribed to W–O, Si–O, and chemisorbed oxygen, respectively. Quantitative fitting analysis indicates that the chemisorbed oxygen content is 9.7%. Such a high proportion of chemisorbed oxygen provides abundant surface active sites for NO_2_ molecules, which serves as the basis for achieving low-temperature response. The W 4f spectrum (Figure 2f) shows peaks at 35.7 and 37.9 eV (W 4f_7_/_2_ and W 4f_5_/_2_, respectively) and at 41.6 eV (W 5p_3_/_2_).

**Figure 3:**
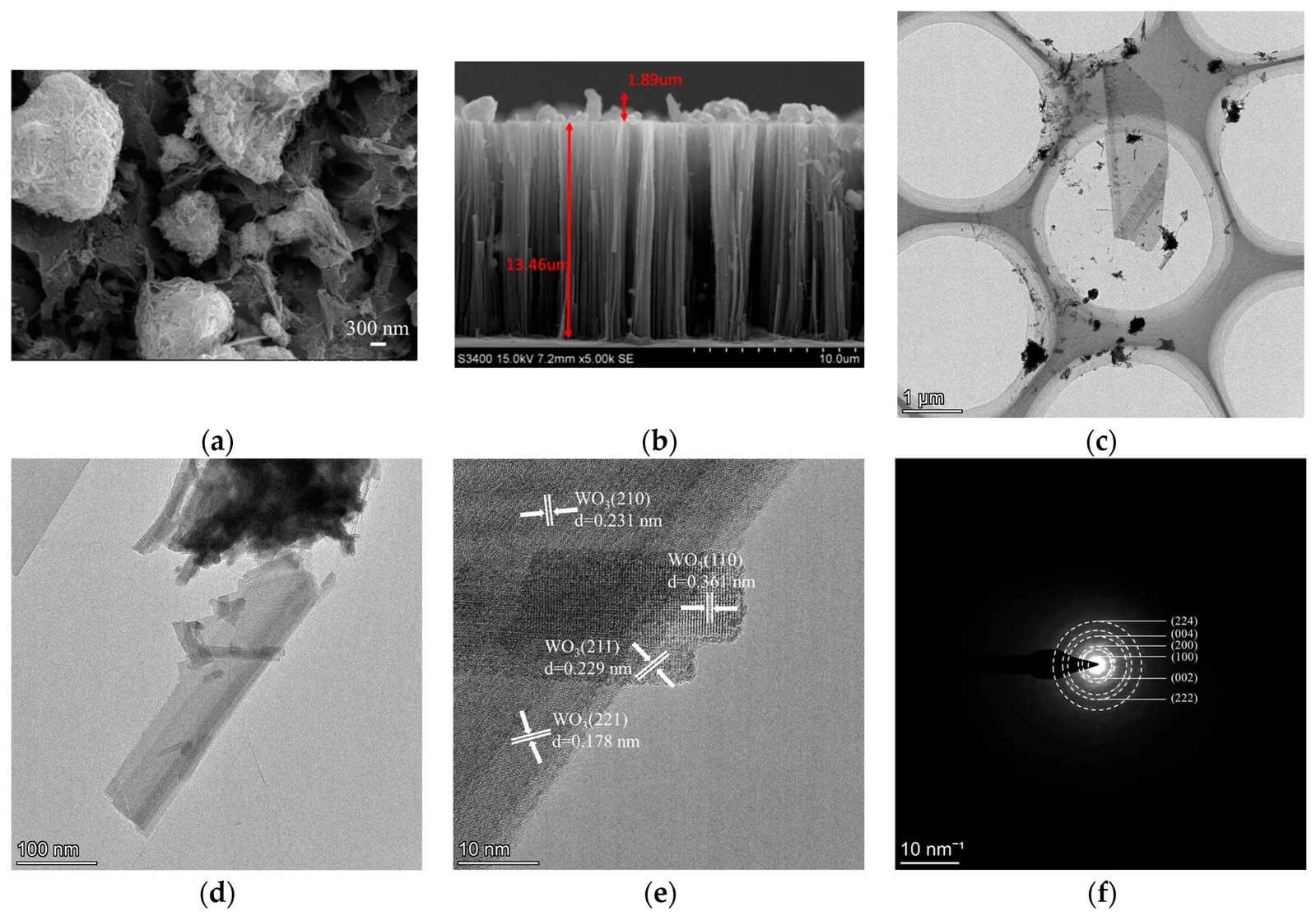
SEM and TEM images of the rGO/WO_3_/SiNWs composite. (**a**,**b**) top-view and cross-sectional SEM images; (**c**,**d**) TEM image; (**e**) HRTEM image; (**f**) SAED pattern.

The morphology of the rGO/WO_3_/SiNWs composite is shown in Figure 3 (SEM and TEM). SEM images at different magnifications (Figure 3a,b) show that the SiNWs are approximately 13.46 μm in length and 130 nm in width, with a porous surface densely covered by wrinkled rGO/WO_3_ films. The rGO/WO_3_ film thickness is approximately 1.89 μm. The WO_3_ nanorods are approximately 729 nm long and 43 nm wide, and are tightly wrapped by rGO to form clusters. As can be seen from the TEM image in Figure 3c,d, a two-dimensional layered material identified as rGO is clearly observed, with WO_3_ and SiNWs fragments attached to its surface. This provides direct evidence of the intimate contact between rGO and WO_3_/SiNWs in the composite. HRTEM analysis (Figure 3e) reveals lattice fringes with spacings of 0.231, 0.361, 0.229, and 0.178 nm, which correspond to the (210), (110), (211), and (221) planes of WO_3_, respectively. The SAED pattern (Figure 3f) displays concentric rings, confirming the high crystallinity of the material. The rings are indexed to the (224), (004), (200), (100), (002), and (222) planes of WO_3_, which is consistent with the hexagonal WO_3_ phase identified by XRD.

**Figure 4:**
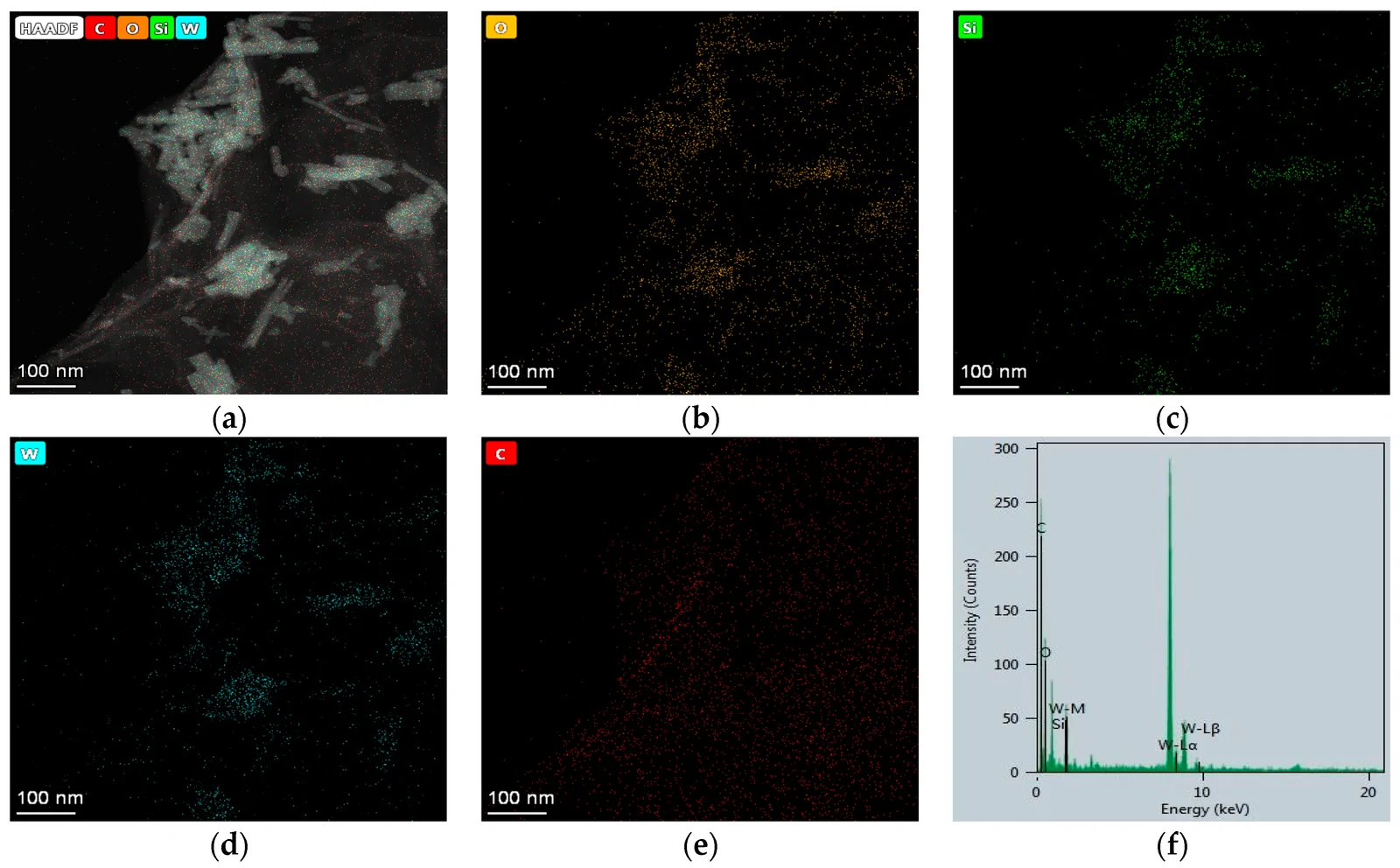
Elemental analysis of the rGO/WO_3_/SiNWs composite. (**a**) HAADF-STEM image; elemental mapping of (**b**) O, (**c**) Si, (**d**) W, and (**e**) C; (**f**) EDS spectrum.

Figure 4 displays the elemental mapping results for the rGO/WO_3_/SiNWs composite. The HAADF image (Figure 4a) and elemental maps (Figure 4b–e) confirm uniform distributions of O, Si, W, and C across the sample. The EDS spectrum in Figure 4f further verifies that only these four elements are present, with no detectable impurities. Combined with the aforementioned characterization results, these observations demonstrate the successful preparation of the ternary composite structure with high purity.

### 3.2. NO_2_ Sensing Performance Evaluation

**Figure 5:**
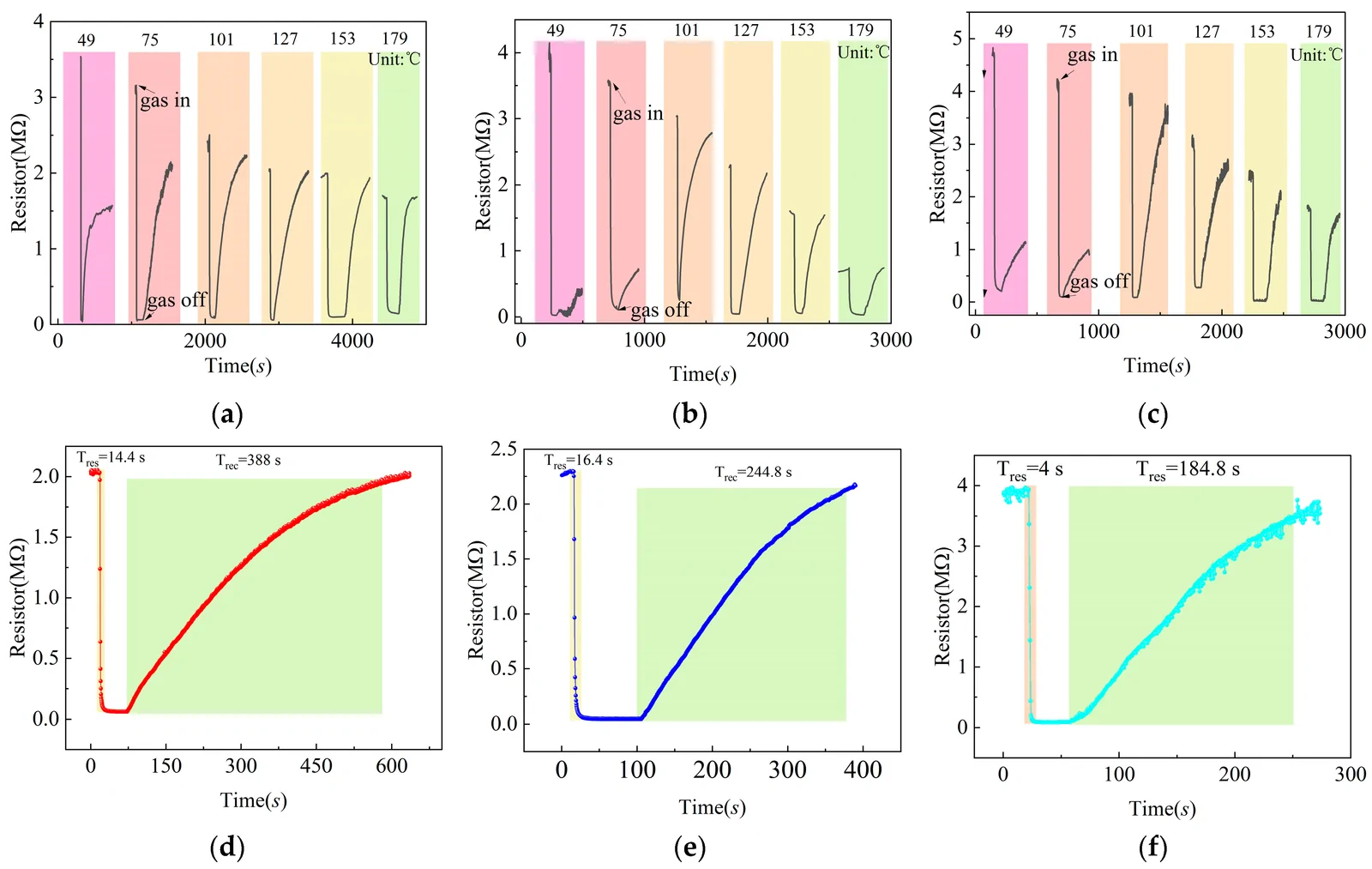
Response and recovery transients of the rGO/WO_3_/SiNWs sensors to 1 ppm NO_2_ at various operating temperatures. (**a**–**c**) Dynamic response/recovery curves of S-1, S-2, and S-3; (**d**–**f**) the response/recovery curve at the optimum operating temperature of S-1, S-2, and S-3.

The responses of each sensor (S-1, S-2, and S-3) to 1 ppm NO_2_ were measured at various operating temperatures, as shown in Figure 5. To clarify the individual contributions of each component and verify the superiority of the ternary system, control samples including pristine SiNWs, WO_3_/SiNWs, rGO/SiNWs, rGO/WO_3_, and WO_3_/SiNWs with 0 wt% rGO were systematically compared for NO_2_ sensing at 1 ppm. Their optimal operating temperatures, sensitivities, and response/recovery times are summarized in Table 1.

As shown in Figure 1 and Table 1, the binary WO_3_/SiNWs sensor exhibited a response of 238, a response/recovery time of 14.8/99.2 s at 127 °C, which was higher than that of pristine SiNWs. For S-1, the optimal sensitivity of 35.3 toward 1 ppm NO_2_ was achieved at 127 °C, with response/recovery times of 14.4 s and 388 s, respectively. When the operating temperature decreased below 101 °C, the response curves could not return to the baseline within the test duration, indicating that the desorption kinetics of NO_2_ molecules were kinetically limited at lower temperatures. For S-2, the highest sensitivity reached 48.3 at 127 °C, with response/recovery times of 16.4/244.8 s; similarly, the desorption process was restricted below 101 °C. For S-3, the optimal sensitivity was 44.7 at 101 °C, with response/recovery times of 4/184.8 s, while the desorption kinetics became insufficient below 75 °C. For the ternary rGO/WO_3_/SiNWs sensors, increasing the rGO content from 0.2 to 2 wt% led to a decrease in sensitivity but a notable reduction in both optimal operating temperature and response/recovery times. The acceleration of recovery was particularly pronounced, which is attributed to the conductive rGO network that facilitates rapid electron transfer and gas desorption. These results demonstrate that the ternary rGO/WO_3_/SiNWs architecture outperforms the binary and single-component counterparts, confirming a synergistic effect among WO_3_, rGO, and SiNWs.

**Figure 6:**
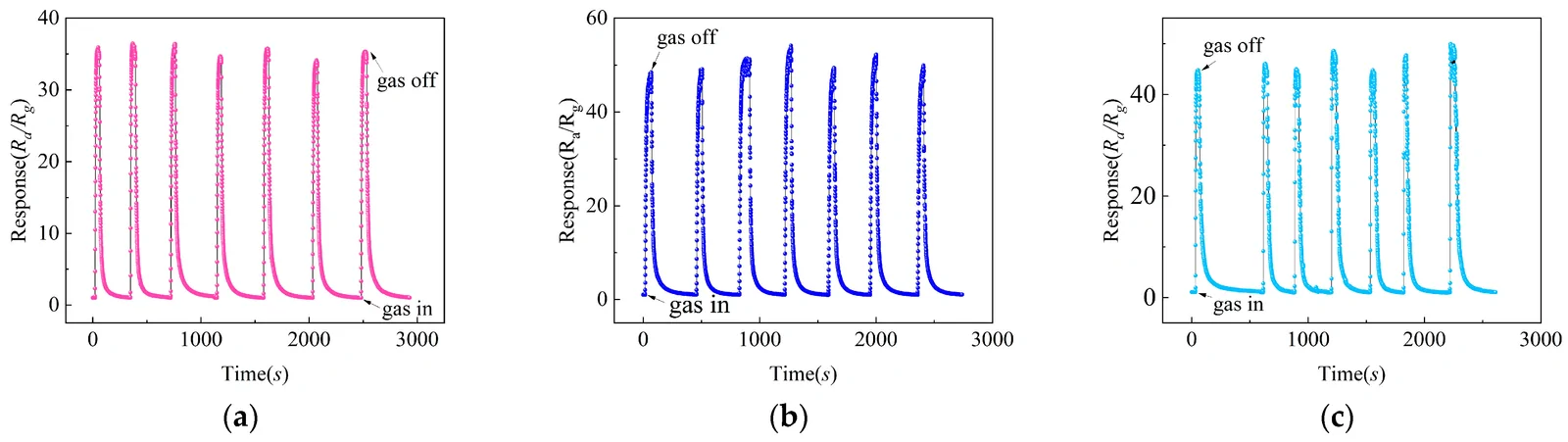
Repeatability tests of the rGO/WO_3_/SiNWs sensors upon seven successive exposures to 1 ppm NO_2_. (**a**) S-1 at 127 °C; (**b**) S-2 at 127 °C; (**c**) S-3 at 101 °C.

The repeatability of the sensors was evaluated by consecutive measurements. As presented in Figure 6a, S-1 was exposed to 1 ppm NO_2_ for seven cycles at 127 °C, yielding sensitivity values of 35.3, 36.2, 35.7, 34.5, 35.6, 34.0, and 35.1, with an RSD of 2.12%. For S-2 under the same operating temperature (Figure 6b), the sensitivities over seven cycles were 48.3, 48.2, 49.5, 51.0, 48.6, 50.9, and 49.1, corresponding to an RSD of 2.36%. For S-3 measured at 101 °C (Figure 6c), the sensitivities were 44.7, 44.5, 44.2, 47.3, 43.9, 47.2, and 48.1, with an RSD of 3.84%. These results demonstrate satisfactory repeatability for all three sensors.

**Figure 7:**
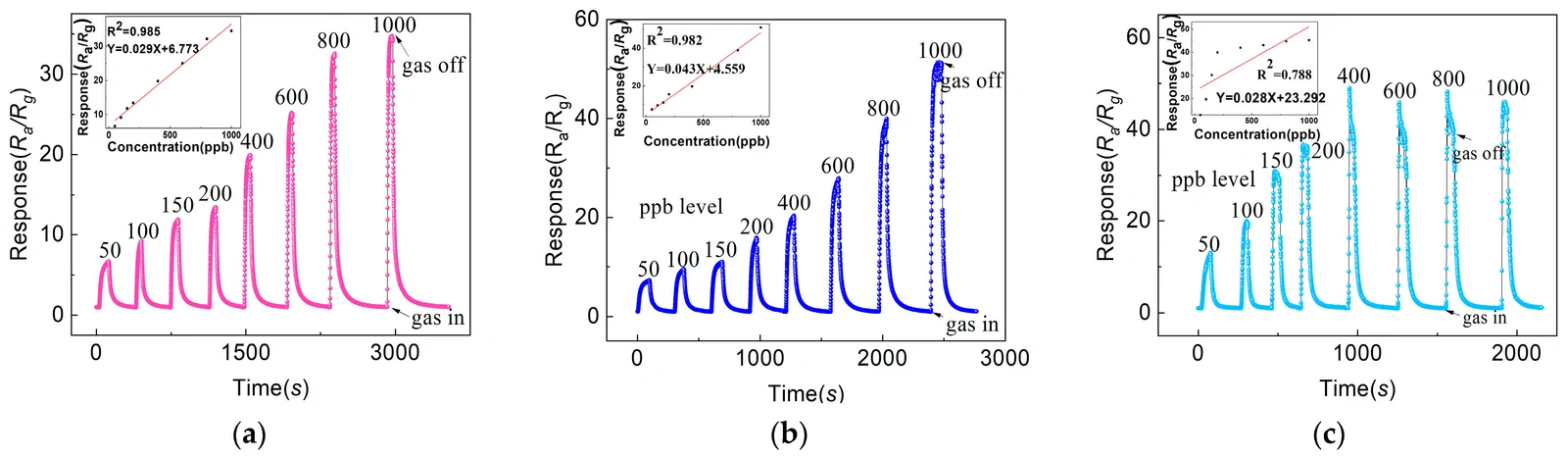
Concentration-dependent responses of the rGO/WO_3_/SiNWs sensors to NO_2_ in the range of 50–1000 ppb. (**a**) S-1; (**b**) S-2; (**c**) S-3. The insets show the corresponding linear fitting curves.

The concentration-dependent responses of S-1, S-2, and S-3 to NO_2_ were examined at their respective optimal operating temperatures over a concentration range of 50–1000 ppb, as shown in Figure 7. For S-1 and S-2, the sensitivity exhibited a good linear relationship with NO_2_ concentration (Figure 7a,b), with fitting coefficients (*R*^2^) of 0.985 and 0.982, and slopes of 0.029 and 0.043, respectively. In contrast, S-3 showed a poorer linearity (*R*^2^ = 0.788) with a slope of 0.028. The reduced linearity for S-3 is likely attributable to the excessive rGO doping, which decreases the number of active sites and facilitates early saturation, thereby hindering the detection at higher NO_2_ concentrations. Nevertheless, S-3 exhibited higher sensitivity toward low-concentration NO_2_ (50 ppb) compared to S-1 and S-2, suggesting its greater potential for detecting NO_2_ at concentrations below 50 ppb.

**Figure 8:**
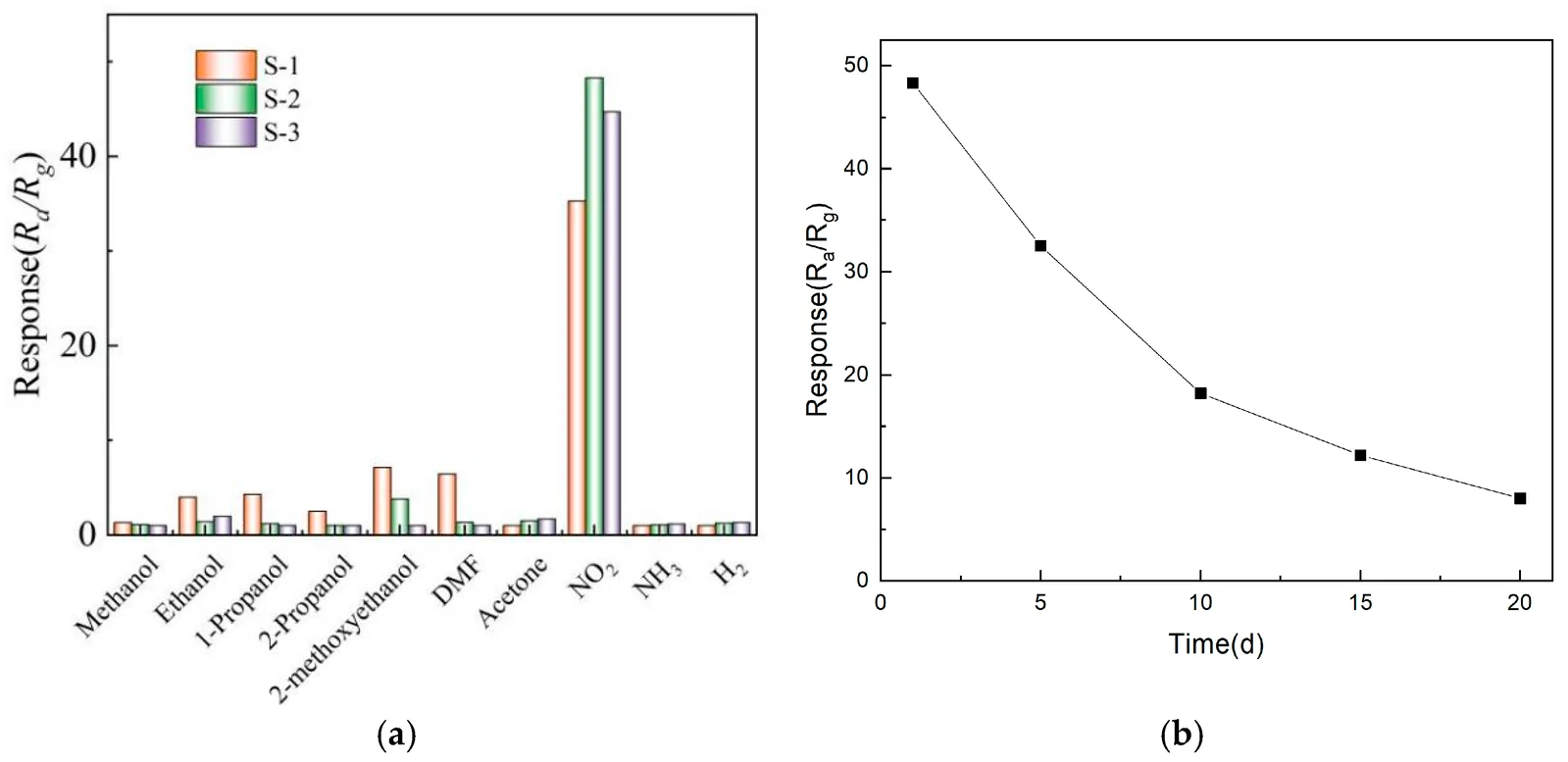
Selectivity and long-term stability of the rGO/WO_3_/SiNWs sensors toward various interfering gases and NO_2_. (**a**) Selectivity; (**b**) long-term stability.

To evaluate the selectivity of S-1, S-2, and S-3, the sensors were exposed to 100 ppm of methanol, ethanol, n-propanol, isopropanol, ethylene glycol monomethyl ether, DMF, acetone, NH_3_, and H_2_, along with 1 ppm NO_2_, at 127, 127, and 101 °C, respectively. All three sensors exhibited the highest response toward NO_2_ among the tested gases (Figure 8a), confirming NO_2_ as the primary target analyte for subsequent investigations.

The long-term stability of the S-2 sensor (0.4 wt% rGO) was measured toward 1 ppm NO_2_ at 127 °C over a period of 20 days under identical conditions. As shown in Figure 8b, the response decreased from 48.3 to 8.0 after 20 days, corresponding to a decline of approximately 83%. This degradation is likely attributed to the gradual oxidation or structural rearrangement of the rGO sheets under continuous thermal and gas exposure, as well as the adsorption of moisture or airborne contaminants on the WO_3_ surface, which passivates the active sites for NO_2_ adsorption.

### 3.3. Gas Sensing Mechanism of WO_3_/SiNWs Heterostructure

**Figure 9:**
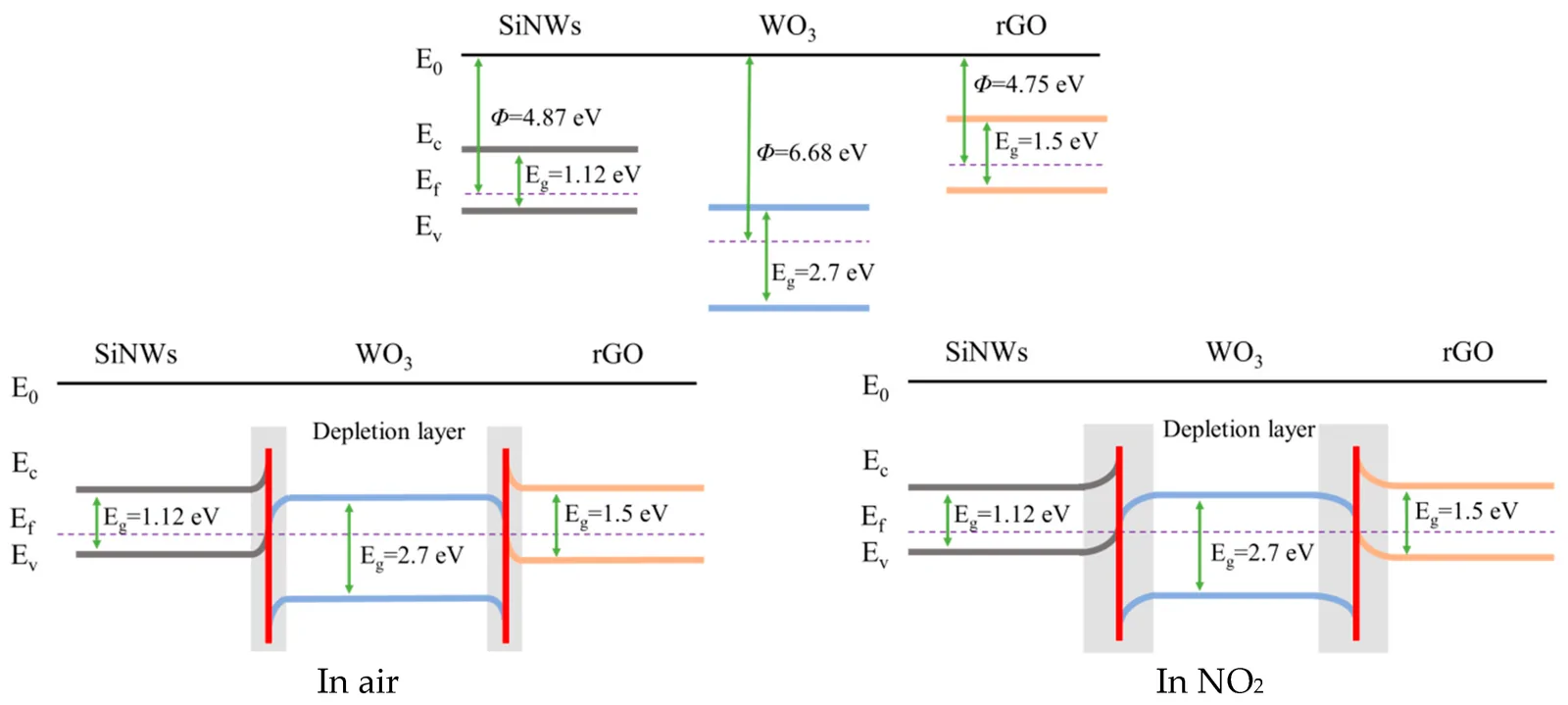
Proposed gas-sensing mechanism of the rGO/WO_3_/SiNWs composite.

The enhanced gas-sensing performance of the rGO/WO_3_/SiNWs composite, as illustrated in Figure 9, originates primarily from the multiple heterojunctions that facilitate electron transfer among WO_3_ nanorods, rGO sheets, and SiNWs. The band gap of WO_3_ is approximately 2.7 eV with a work function of about 6.68 eV. Depending on the reduction method, the band gap of rGO ranges from 1 to 1.69 eV [25]. The p-type SiNWs exhibit a band gap of roughly 1.12 eV and a work function of about 4.87 eV. Upon contact, p-n heterojunctions are proposed to form at both the WO_3_/rGO and WO_3_/SiNWs interfaces based on the band alignment of the constituent materials and the observed sensing behavior. Driven by the carrier concentration gradients across these interfaces, holes from p-type rGO and SiNWs migrate toward n-type WO_3_, while electrons from n-type WO_3_ flow toward SiNWs and rGO. This charge transfer leads to upward band bending and the formation of interfacial depletion layers.

Under ambient conditions, oxygen molecules are chemisorbed on the surface and capture electrons from the conduction band of WO_3_ and holes from the valence bands of rGO and SiNWs, generating reactive oxygen species such as O_2_^−^. Recent defect-engineering studies on MOS gas sensors have shown that interfacial oxygen vacancies can significantly promote the adsorption and generation of such reactive oxygen species, thereby amplifying the sensing response. [7] In the rGO/WO_3_/SiNWs system, the multiple heterojunctions and the high proportion of chemisorbed oxygen (9.7%) play an analogous role: the interfaces provide abundant active sites for oxygen adsorption, and the chemisorbed oxygen species serve as the primary reaction sites for NO_2_ detection.

When exposed to NO_2_, the gas molecules extract electrons from the composite surface and inject holes into rGO and SiNWs. As an n-type semiconductor, WO_3_ experiences electron depletion, leading to a widened depletion layer. In contrast, rGO and SiNWs, being p-type, exhibit an increased hole concentration, which enhances the hole accumulation layers at the interfaces, making holes the dominant carriers in the near-interface regions. The reduced carrier concentration gradient across the interfaces intensifies the band bending, which enhances carrier mobility and thereby improves the overall gas-sensing response [26]. In this ternary system, WO_3_ provides specific adsorption sites for NO_2_, rGO serves as a conductive channel that accelerates charge transfer and reduces the operating temperature, and SiNWs offer a large specific surface area scaffold along with a conductive pathway compatible with CMOS technology.

## 4. Conclusions

A ternary rGO/WO_3_/SiNWs composite structure was fabricated by combining metal-assisted chemical etching, hydrothermal synthesis, and spin-coating. XRD, XPS, SEM, TEM, and EDS analyses confirm that hexagonal WO_3_ nanorods and rGO sheets form intimately contacted and stable multiple heterojunctions on the SiNWs surface.

The rGO content strongly influences the sensing behavior. The 0.4 wt% sample (S-2) exhibits a response of 48.3 to 1 ppm NO_2_ at 127 °C, with response/recovery times of 16.4/244.8 s. Increasing the rGO content to 2 wt% (S-3) lowers the optimal temperature drops to 101 °C and shortens the response/recovery times to 4/184.8 s, while the response remains 44.7. S-1 and S-2 show a good linear relationship between sensitivity and NO_2_ concentration in the range of 50–1000 ppb. Excessive rGO doping (2 wt%) reduces the linearity at higher concentrations but markedly enhances the sensitivity toward low-concentration NO_2_ (≤50 ppb).

Repeatability and selectivity tests confirm the stable and preferential recognition of NO_2_ by the ternary system. The enhanced gas-sensing mechanism is attributed to the synergistic modulation of interfacial depletion layers and carrier transport through the multiple p–n heterojunctions among WO_3_, rGO, and SiNWs, which effectively amplify the resistance change induced by gas adsorption. This work provides a design strategy for heterojunction engineering of low-power NO_2_ sensors.

Future work will focus on improving the stability through surface encapsulation and optimization of the annealing conditions.

## Figures and Tables

**Table 1:** Comparison of NO_2_ sensing performance (1 ppm) for control samples and ternary composites.

Samples	Initial GO/WO_3_ Mass Ratios (wt%)	Working Temperature (°C)	Response (*R*_g_/*R*_a_)	Response Time	Recovery Time (s)
SiNWs	-	153	5.4	149	227
WO_3_/SiNWs	-	127	238	14.8	99.2
rGO/SiNWs	-	127	4.8	125	189
WO_3_	-	400	1.48	15	36
rGO/WO_3_	2	328	1.20	16	32
WO_3_/SiNWs	-	127	238	14.8	99.2
rGO/WO_3_/SiNWs (S1)	0.2	127	35.3	14.4	388
rGO/WO_3_/SiNWs (S2)	0.4	127	48.3	16.4	244.8
rGO/WO_3_/SiNWs (S3)	2	101	44.7	4	184.8

## Data Availability

The data that support the findings of this study are available from the corresponding author upon reasonable request.
